# Comparison of minimal detectable protoporphyrin IX concentrations with a loupe device and conventional 5-ALA fluorescence microscopy: an experimental study

**DOI:** 10.1117/1.JBO.28.10.106004

**Published:** 2023-10-31

**Authors:** Mario Mischkulnig, Denise Traxler, Lisa I. Wadiura, Alexandra Lang, Matthias Millesi, Barbara Kiesel, Georg Widhalm

**Affiliations:** a Medical University of Vienna, Department of Neurosurgery, Vienna, Austria; b Medical University of Vienna, Department of Oral and Maxillofacial Surgery, Vienna, Austria

**Keywords:** 5-aminolevulinic acid, fluorescence, loupe device, protoporphyrin IX, detectable concentration

## Abstract

**Significance:**

The 5-aminolevulinic acid (5-ALA) fluorescence technique is now widely applied for intraoperative visualization of specific central nervous system (CNS) tumors. Previous technical implementations of this technique have relied on specifically modified surgical microscopes to visualize intratumoral fluorescent protoporphyrin (PpIX). While this approach evidently allows for reliable intraoperative tumor visualization, it requires the availability of specifically modified surgical microscopes and their use even in cases where the operating neurosurgeon would prefer to use surgical loupes. Recently, a novel loupe device was introduced that is also capable of visualizing 5-ALA fluorescence.

**Aim:**

The aim of this study was therefore to compare the detected PpIX concentrations between the conventional fluorescence microscope and the novel loupe device.

**Approach:**

We used fluorescence phantoms of different PpIX concentrations for comparison between a conventional fluorescence microscope and the novel loupe device. For this purpose, we created fluorescence images using the excitation light sources of the conventional fluorescence microscope and the loupe device with both available background illumination modes (low and high). Subsequently, the minimal detectable PpIX concentrations according to each technique were determined by five independent neurosurgeons.

**Results:**

Using the conventional fluorescence microscope, the median minimal detectable PpIX concentration was 0.16  μg/ml (range: 0.15 to 0.17  μg/ml). By the loupe device, the median minimal detectable PpIX concentration was 0.12  μg/ml (range: 0.10 to 0.12  μg/ml) and 0.08  μg/ml (range: 0.07 to 0.08  μg/ml) for the high- and low-modes, respectively. Altogether, the minimal detectable PpIX concentrations were significantly lower using the loupe device compared to the conventional fluorescence microscope (p=0.007).

**Conclusions:**

Our data indicate that the novel loupe device is able to visualize 5-ALA fluorescence with high sensitivity and thus might serve as a powerful tool for visualization of specific CNS tumors in the future.

## 1 Introduction

Neurosurgical resection constitutes the initial treatment modality in most brain tumor entities and is thus crucial to improve the prognostic outcome in these patients.^1^^–^^3^ While maximal safe resection is generally the surgical aim, an incomplete resection is a frequent pitfall in the surgical treatment of brain tumors due to poor intraoperative tumor visualization.^4^ A number of intraoperative tumor visualization techniques have been implemented in the neurosurgical routine, whereas fluorescence guidance with the orally administrable fluorescent dye 5-aminolevulinic acid (5-ALA) represents one of the most powerful methods.^4^^–^^6^ After oral ingestion, 5-ALA is metabolized by enzymes of the heme biosynthesis pathway to fluorescent protoporphyrin IX (PpIX) that emits a characteristic red fluorescence signal with a peak at 635 nm after peak excitation with light-sources in the violet spectrum.^7^^–^^9^ Since the initial application of 5-ALA induced fluorescence for brain tumor surgery in 1998, this innovative technique has shown a promising diagnostic accuracy in specific central nervous system (CNS) tumors and now constitutes a standard of care in the surgical treatment especially of high-grade gliomas at many centers worldwide.^10^^–^^12^

Intraoperative 5-ALA fluorescence is commonly visualized with assistance of a specifically modified neurosurgical microscope that is equipped with a certain module for fluorescence visualization.^13^^,^^14^ This module typically consists of a xenon light source in conjunction with a 440 nm short-pass filter to provide violet-blue excitation light as well as a 440 nm long-pass filter added to the observer light path to selectively visualize the fluorescence signal.^15^ Interestingly, some variance between illumination light intensities has been described even between different established microscope models.^16^ While this technical implementation is meanwhile well established and has been shown to allow clinically reliable tumor detection, fluorescence visualization is limited to cases where a surgical microscope is used.^17^^–^^19^ However, many neurosurgeons prefer the use of a surgical loupe device for improved intraoperative tumor visualization instead of a surgical microscope and thus the conventional fluorescence visualization technique cannot be applied.^19^^,^^20^ Further, modified neurosurgical microscopes necessary for fluorescence visualization are not available at every neurosurgical center.^19^

Recently, a specific loupe device consisting of a headlight and loupe spectacles tailored to facilitate intraoperative 5-ALA induced fluorescence visualization was successfully introduced to the neurosurgical field.^15^ Instead of a filtered xenon light source typically utilized in the modified surgical microscope, this device relies on light-emitting diodes (LED) with wavelengths of 409 and 450 nm to provide excitation light.^15^ First clinical observations demonstrated a successful implementation of this novel loupe device with a sufficient fluorescence signal.^21^ In addition, a further study found that a subset of specimens could only be visualized by fluorescence with the loupe device, but demonstrated absence of fluorescence with the conventional fluorescence microscope.^15^ This study also demonstrated a slightly higher optical power density in fluorescence visualization mode at 45 cm working distance of $6.4\;\mathrm{mW}/{\mathrm{cm}}^{2}$ for the loupe device compared to $5.1\;\mathrm{mW}/{\mathrm{cm}}^{2}$ with a Zeiss Kinevo 900 microscope at 35 cm working distance.^15^ Likewise, a video case report demonstrated substantially improved fluorescence visualization with the loupe device compared to a modified surgical microscope.^22^ At present, a systematical comparison of PpIX concentrations between this novel loupe device and a routinely applied modified surgical microscope for conventional fluorescence detection is missing in the current literature.

The aim of our study was thus to compare differences in minimally detectable PpIX concentrations analyzed by the loupe device and a modified neurosurgical microscope as routinely used in fluorescence guided surgery according to five independent raters.

## 2 Material and Methods

In this study, we examined differences in PpIX fluorescence detection between a newly developed loupe device system and a frequently applied modified neurosurgical microscope for conventional fluorescence visualization. Since no patients or patient data were included in this study, ethical approval was waived in accordance with the guidelines of ethics committee at the Medical University of Vienna.

### 2.1 Fluorescence Visualization Devices

**Fig. 1:**
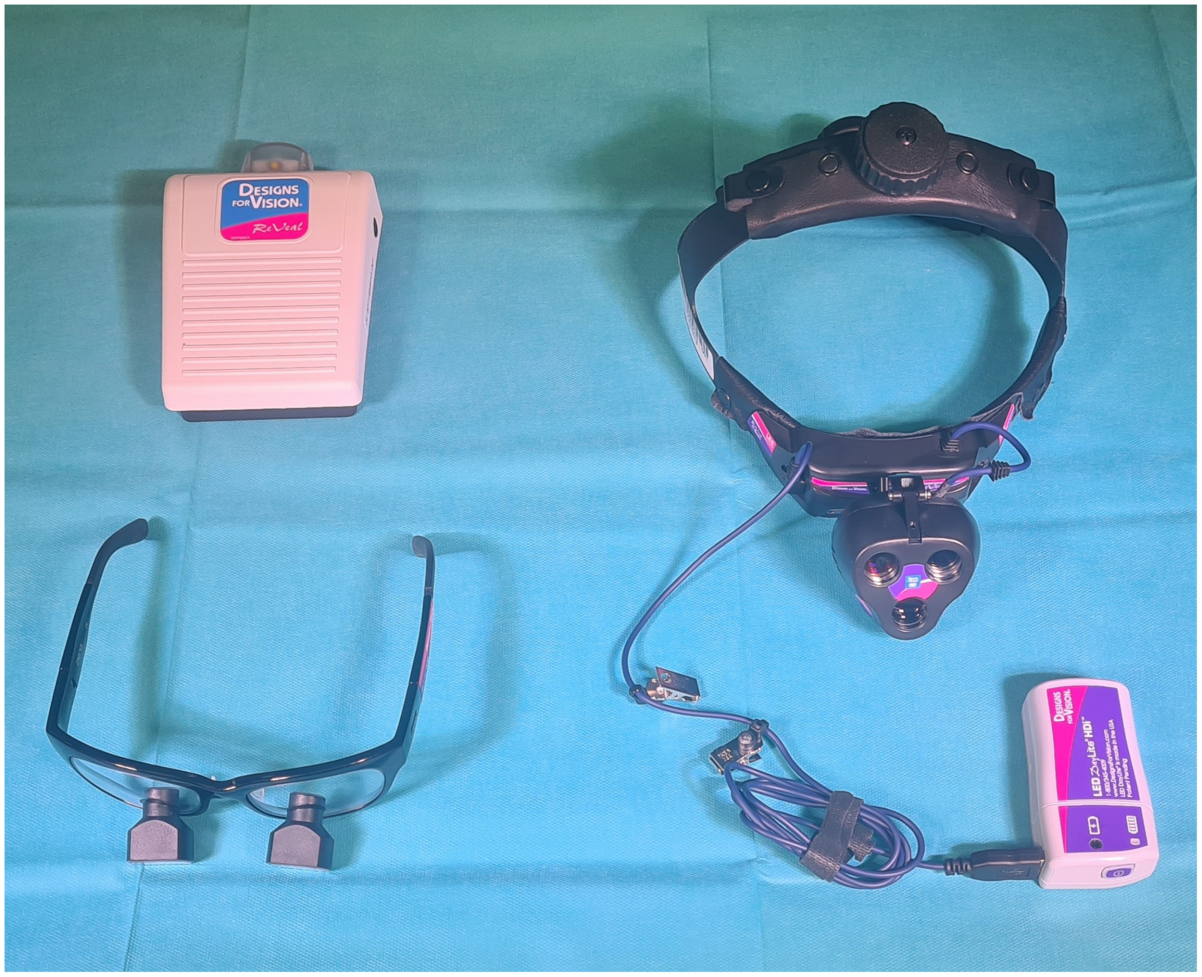
Illustration of the loupe device used within this study. All essential intraoperative components of the novel loupe device are shown. The specifically developed headlight (top right) is wired to a rechargeable battery pack (bottom right). The remote paddle (top left) allows the surgeon to easily toggle between illumination modes, while the surgical goggles (bottom left) feature specific filters that facilitate conventional white light surgery as well as adequate and safe visualization of 5-ALA induced fluorescence.

For routine fluorescence visualization, a Zeiss Kinevo 900 (Carl Zeiss AG, Oberkochen, Germany) surgical microscope with two 300 w xenon light sources and included Blue-400 module for fluorescence guided surgery was used. As loupe device, the meanwhile commercially available ReVeal fluorescence guided surgery system (Designs for Vision Inc., Bohemia, New York) was investigated. Besides a surgical loupe with specific filters for fluorescence visualization, the loupe device features a headlight that facilitates 5-ALA fluorescence excitation in addition to the conventional white light illumination. For fluorescence visualization, the headlight features two distinct LED light sources of 409 and 450 nm wavelength.^15^ While 5-ALA fluorescence excitation is primarily induced by the 409 nm light source, the 450 nm light source is primarily aimed at providing background illumination and minimizing the effect of tissue autofluorescence rather than exciting PpIX fluorescence.^15^ To optimize the background illumination for different intensities of 5-ALA induced fluorescence signal, the headlight features two specific modes specifically the “low” and “high” background illumination modes that correspond to currents of 2 and 5 mA applied to the 450 nm LED, respectively.^15^ Furthermore, a rechargeable battery pack connected to the headlight source as well as a wireless foot pedal allowing the surgeon to easily switch between three illumination modes (white light/fluorescence—low mode/fluorescence—high mode) are further important components of the reveal fluorescence guided surgery system. The loupe device with all essential components used in this study is shown in Fig. 1.

### 2.2 Production of Phantoms

We used fluorescence specimens (fluorescence phantoms) of known PpIX concentrations (258385, Sigma-Aldrich, Merck, St. Louis, Missouri) that were created in a solution of 99.5% phosphate buffered saline pH 7.4 (10010015, Gibco, Thermo Fisher Scientific, Waltham, Massachusetts). Furthermore, 0.5% Tween20 (P9416, Sigma-Aldrich Sigma-Aldrich, Merck, St. Louis, Missouri) was used as emulsifying agent as previously identified as essential to create stable aqueous solutions.^23^ According to previously published data on PpIX tissue concentrations detectable with a modified surgical microscope from our own group as well as Suero Molina et al., minimal visible PpIX concentrations were expected to be observed in the concentration range between 0.05 and 1.50  μg/ml.^24^^,^^25^ A preliminary test series was thus performed and determined visible PpIX fluorescence to be detectable between 0.05 and 0.10  μg/ml (loupe device, low mode), 0.10 to 0.15  μg/ml (loupe device, high mode), and 0.15 to 0.20  μg/ml (modified microscope). According to this observation, 15 ml liquid phantoms with PpIX concentration increases of 0.01  μg/ml per phantom ranging from 0.01 to 0.30  μg/ml were created in Falcon 15 ml high-clarity polypropylene conical tubes (352096, Corning, New York) and were subsequently vortexed before each analysis in order to ensure consistent PpIX concentrations across the phantoms.

### 2.3 Fluorescence Analysis

Fluorescence images were obtained under standardized conditions in a darkened operating room from the fluorescence phantoms of different PpIX concentrations (0.01 to 0.30  μg/ml) with assistance of the excitation light sources of either the modified surgical microscope or the loupe device and using the integrated standard three-chip HD camera of the Zeiss Kinevo 900 system. With regard to the loupe device, we used both background illumination modes (low and high) separately. The fluorescence phantoms were placed at a distance of ∼400  mm in accordance with the focal length of the surgical goggles supplied with the loupe device in order to ensure realistic examination conditions. The light intensity of the microscope light source was set to 100% with activated focus light link and auto brightness settings in order to apply the maximum safely applicable dose as routinely performed during surgery while the predetermined setting of each loupe device mode was used. The same phantoms were used for the analyses with all three light sources and images were first taken with the microscope followed by the low background illumination mode and lastly the high background illumination mode of the loupe device. In order to prevent relevant photobleaching of the phantoms, exposure times were minimized to an average of <18.0  s (microscope light source), 14.46 s (loupe—strong background illumination mode), and 14.6 s (loupe—weak background illumination mode). Examination was performed according to color images as established in routine applications on the standard 24″ HD video touchscreen screen of the Zeiss Kinevo 900 system. Subsequently, five neurosurgeons with large experience in fluorescence procedures were asked to independently assess the fluorescence images in order to identify the fluorescence phantom with the lowest PpIX concentration for each modality (modified microscope and loupe device low/high mode) in which they could with certainty discern a fluorescence signal.

### 2.4 Statistical Analysis

Statistical testing as well as figure production was performed using SPSS statistical software (Version 27.0, IBM Inc., Armonk, New York). Initially, the presence of normal distribution was analyzed for each fluorescence detection technique using the Shapiro–Wilk test. Descriptive statistics included characterization of fluorescence rating with mean ± standard deviation in case normal distribution was present for all analyses and median (range) in case standard deviation was not present for the rating with at least one technique. Likewise, inferential statistical analyses were performed using the repeated measures ANOVA/paired t-test in case of normal distribution while otherwise the Friedman test/paired samples Wilcoxon test were used for the analysis of all three and distinct pairs of the visualization techniques, respectively.

## 3 Results

In this study, we compared the minimally detectable PpIX concentrations investigated by either a loupe device or a routinely used modified neurosurgical microscope for conventional fluorescence detection. The underlying fluorescence rating data of five neurosurgeons for all three illumination light sources are provided in Table S1 in the Supplementary Material.

### 3.1 Overall Fluorescence Signal Assessment

**Fig. 2:**
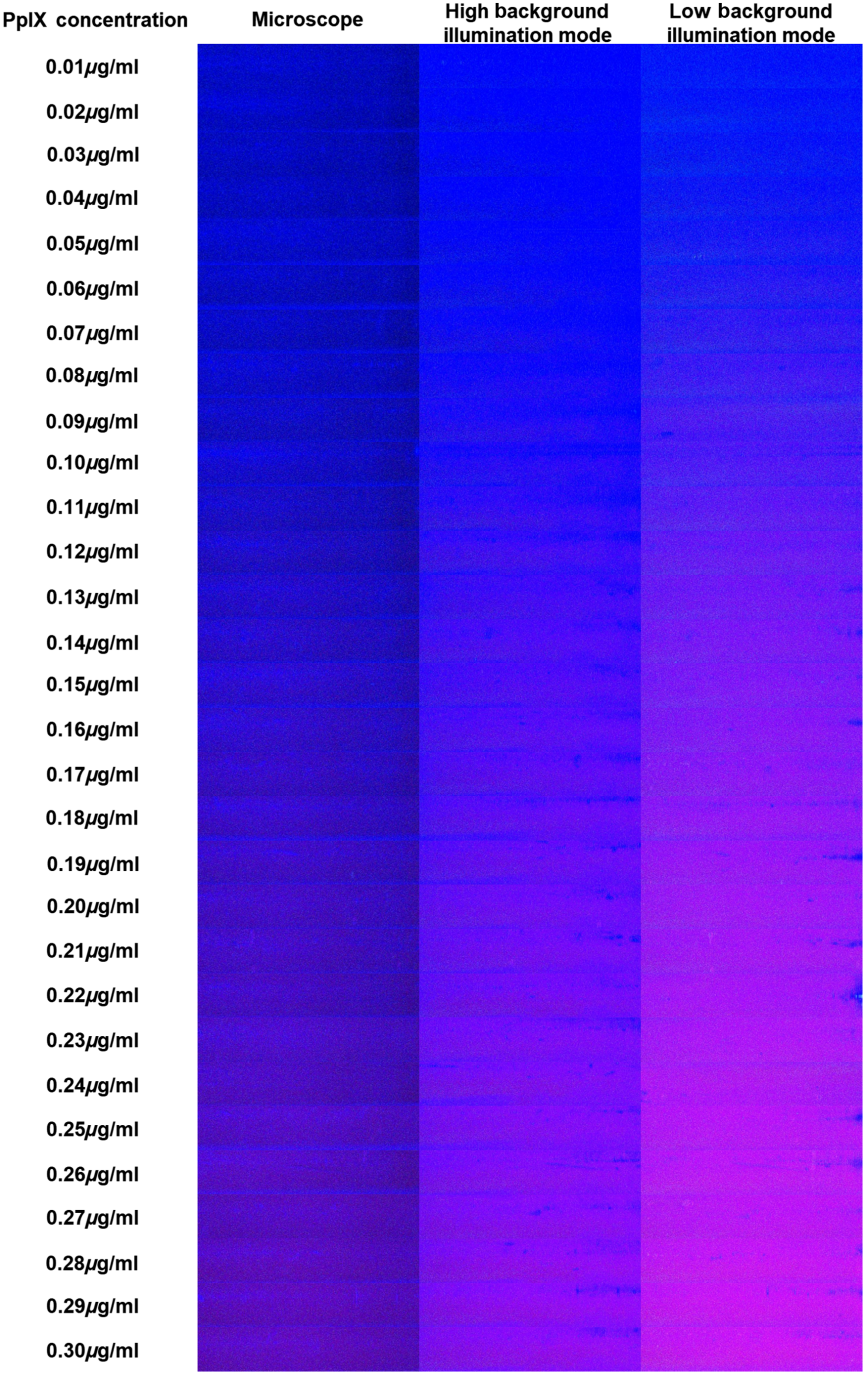
Images of fluorescence phantoms with different excitation light sources. Fluorescence phantoms with ascending PpIX concentrations of 0.01  μg/ml (top) to 0.30  μg/ml (bottom) as visualized with the conventional fluorescence microscope light source (left) and the high (middle) and low (right) background illumination mode of the loupe device are shown. Images were cropped to only include the fluorescence phantoms but where not subject to any processing that might impact signal intensity. Stronger fluorescence intensity is clearly visible with the loupe device light source in the high and particularly low background illumination mode as compared to the microscope light source.

Overall, all ratings of the five observers according to both loupe device background illumination modes (low and high mode) as well as the modified fluorescence microscope determined PpIX concentrations between 0.07 and 0.17  μg/ml for the minimal recognizable fluorescence signal. Testing for normal distribution across all raters with the Shapiro–Wilk test verified the presence of a normal distribution only for ratings with the microscope (p=0.314) but rejected it for both loupe device modes (p<0.0005 and p=0.046, respectively). Therefore, we used non-parametric tests for the subsequent statistical analyses. Illustrative images of the fluorescence phantoms with ascending PpIX concentrations of 0.01 to 0.30  μg/ml as visualized with microscope and the loupe device (low and high modes) are shown in Fig. 2.

### 3.2 Minimal Recognizable Fluorescence Signal: Conventional Fluorescence Microscope and Loupe Device

**Fig. 3:**
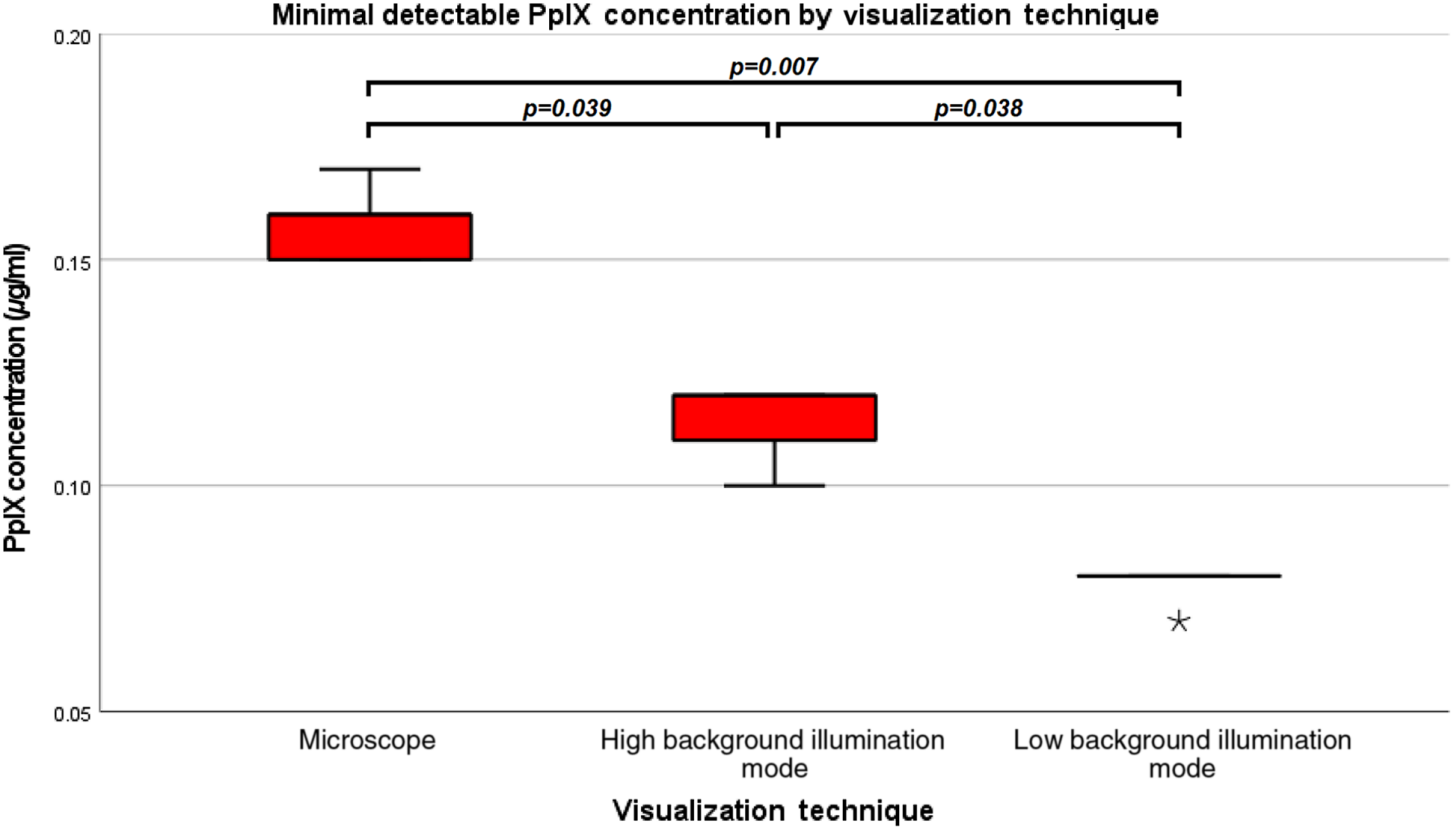
Minimal detectable PpIX concentrations by visualization technique. Boxplot diagram of minimal detectable PpIX concentrations by the conventional fluorescence microscope and the loupe device in high and low background illumination modes are shown. The minimal detectable PpIX concentration was significantly higher with the conventional fluorescence microscope compared to the loupe device and lower concentrations could be detected with the low background illumination mode as compared to the high background illumination mode. *Characterizes an outlier within the low background illumination mode with a rating of 0.07  μg/ml.

According to the rating of five independent observers, phantoms with the minimal reliably discernable fluorescence signal according to the modified fluorescence microscope showed a median PpIX concentration of 0.16  μg/ml (range: 0.15 to 0.17  μg/ml). By analysis with the loupe device, the minimal recognizable fluorescence signal showed a median PpIX concentration of 0.12  μg/ml (range: 0.10 to 0.12  μg/ml) for the low mode and 0.08  μg/ml (range: 0.07 to 0.08  μg/ml) for high mode. We found a significant difference in the minimal detectable PpIX concentrations between conventional fluorescence microscope and the loupe device with the low and high modes (p=0.007). In detail, subgroup analyses demonstrated significant differences between the conventional fluorescence microscope and the high loupe device mode (p=0.039) as well as the low loupe device mode (p=0.042) and between the two loupe device modes (p=0.038). Boxplot diagrams of minimal detectable PpIX concentrations by the conventional fluorescence microscope and the loupe device are provided in Fig. 3.

### 3.3 Variability of Minimal Reliably Detectable Concentrations Across and Within Visualization Techniques

Since there is inherent interobserver variability associated with any observer-based fluorescence assessment, we compared the percent variability between the different observers according to both fluorescence visualization techniques. According to our data, the variability of the minimal detectable PpIX concentrations between the five different raters was 0.02  μg/ml (13.3%) for the assessment with the microscope as compared to 0.01  μg/ml (14.3%) for the analysis with the high loupe device mode and 0.02  μg/ml (20%) for the low loupe device mode. Compared to this modest variability between observers within each visualization technique, markedly larger relative differences between the different visualization techniques were observed. In detail, the minimal detectable PpIX concentration using the microscope was 0.04  μg/ml (33.3%) higher compared to the high loupe device mode and even 0.08  μg/ml (100%) higher compared to the low loupe device mode.

## 4 Discussion

Throughout the last 20 years, 5-ALA induced fluorescence has been established as powerful intraoperative tumor visualization technique in neurosurgery. While fluorescence detection is typically performed with the assistance of specifically equipped neurosurgical microscopes, development of different loupe devices for fluorescence visualization has been documented since 2013 and recently resulted in the clinical admission of the first device capable of detecting 5-ALA fluorescence without the use of surgical microscopes.^4^^,^^15^^,^^21^^,^^26^ Interestingly, the first clinical investigation suggests a higher sensitivity of the meanwhile commercially available loupe device for 5-ALA induced fluorescence as compared to the conventional fluorescence microscope.^15^ In detail, a study in 240 specimens from 26 patients demonstrated a sensitivity of 100% and specificity of 95% for the fluorescence rating according to the loupe device compared to conventional fluorescence microscopy.^15^ Interestingly, 14 specimens could be visualized only with the loupe device, but not with conventional fluorescence microscopy.^15^ In another series of 11 patients, there was 100% accordance between the loupe device and conventional fluorescence microscopy with regard to presence of a fluorescence signal, although the authors noted the fluorescence signal tended to be more vivid using the loupe device.^21^ According to initial clinical observations as well as first cases documented in the literature, there is clear evidence that the investigation with the loupe device may result in a stronger and more vivid fluorescence signal compared to conventional fluorescence microscopes.^22^ Since a systematical comparison of detectable PpIX concentrations between this novel loupe device and a routinely applied conventional fluorescence microscope is missing, we performed this current experimental study to compare minimal detectable PpIX concentrations with both techniques.

### 4.1 Present Study

In the present study, we thus compared the minimally detectable PpIX concentrations analyzed by the loupe device and a conventional fluorescence microscope by using images of fluorescence phantoms with increasing PpIX concentrations. Subsequently, the minimal PpIX concentrations associated with a definitely recognizable fluorescence signal for both techniques were determined according to five independent raters. According to our data, the minimal detectable PpIX concentrations were significantly lower using the loupe device compared to the conventional fluorescence microscope. To our knowledge, this is the first systematical experimental analysis demonstrating that the novel loupe device is capable of detecting lower PpIX concentrations than the conventional microscope. Consequently, this novel loupe device represents a promising diagnostic tool for intraoperative 5-ALA fluorescence detection during brain tumor surgery. While the high background illumination mode of the loupe device was capable of visualizing PpIX concentrations 25% lower than the fluorescence microscope, the 5-ALA signal was consistently stronger with the low background illumination mode resulting in detectable PpIX concentrations 50% lower compared to the conventional fluorescence microscope.

### 4.2 Clinical Relevance

According to the findings of this study, the novel loupe device constitutes easily applicable alternative to surgical microscopes for 5-ALA fluorescence guided surgery and thus might be useful for resection especially of high-grade gliomas. As previously pointed out by authors of similar studies, however, it is important to emphasize that the translation of absolute PpIX concentration values from experimental analysis in PpIX solutions to tissue concentrations is not simply possible since a number of variables need to be considered.^24^ In this sense, tissue factors as well as specific distances and angles between the investigated area and the microscope/loupe device in real life settings may oftentimes differ from those present in our experiments.^27^ The presence of autofluorescence as well as fluorescence absorbents in particular is a major challenge in the translation of *in-vitro* detected fluorescent PpIX concentrations to *in-vivo* observed fluorescence behavior.^15^^,^^23^^,^^27^^,^^28^ It is therefore important to note that the absolute PpIX concentrations identified in this study are primarily valid for the specific chosen methodology and can not necessarily be directly translated to absolute PpIX concentrations in tumor tissue. Future investigations in animal models and patient specimens should thus be performed to investigate fluorescence differences on a tissue level. In contrast, however, our results provide clear and valid evidence that the loupe device can detect PpIX concentrations up to 50% lower than routinely used surgical microscopes.

The loupe device may therefore not only constitute a promising alternative for neurosurgeons preferring the use of a surgical loupe device instead of a surgical microscope or for centers without the availability of modified neurosurgical microscopes but also allow PpIX fluorescence detection with improved sensitivity resulting in the visualization of previously not detectable tumor tissue in certain cases. In this sense, the low background illumination mode in particular is a promising tool for the visualization of previously not reliably detectable tumors with the conventional technique, such as low-grade gliomas and brain metastases. Since it is currently not clear to which extent the improved fluorescence signal may result in increased false-positive rates, a systematical correlation of tissue samples with presence and absence of visible fluorescence according to the loupe device with histopathological characteristics in a large patient series should be performed.

### 4.3 Limitations

The following limitations should be considered in the interpretation of the results presented in this study: First, the aim of this study was to establish differences in detectable PpIX concentrations between the loupe device and a conventional fluorescence microscope through an experimental approach, no histopathological correlations are available in this study. While a higher sensitivity of the loupe device for PpIX fluorescence was clearly demonstrated, no conclusions can thus be drawn as to whether this translates to increased rates of true positive and/or false positive fluorescence ratings according to histopathological correlations. Second, this investigation solely focused on the PpIX fluorescence visualization capabilities of both studied devices and did not compare other important factors for routine clinical use such as ergonomics and usability. Future studies outlining model procedures and routine clinical use of the novel loupe device will therefore be crucial to identify other strengths and weaknesses of the device and identify the most suitable device for distinct applications accordingly. Third, the same phantoms were used for analyses with all three excitation light sources. Given the sequence of analyses performed in this study, a slight bias resulting in underestimating the differences between the microscope and loupe device and overestimating the differences between both loupe device modes due to photobleaching may be present. While exposure times were minimized to prevent relevant photobleaching according to previous studies and the main observation of a significant difference between the microscope and loupe device was validly established, the difference between both loupe device modes should to be validated in future examinations.

## 5 Conclusions

In this first experimental investigation to date, we performed a systematical analysis of minimal detectable PpIX concentrations with a novel loupe device in comparison with conventional surgical fluorescence microscopy in a series of fluorescence phantoms. According to our data, the minimal detectable PpIX concentrations were significantly lower using the loupe device compared to the conventional fluorescence microscope. Consequently, this novel loupe device might be used as powerful alternative to conventional fluorescence microscopes for improved visualization during surgery of specific CNS tumors in the future.

## Data Availability

All data in support of the findings of this paper are available within the article or as the Supplementary Material.
