# Supplementary material for: Comparison of minimal detectable protoporphyrin IX concentrations with a loupe device and conventional 5-ALA fluorescence microscopy: an experimental study
Source: J Biomed Opt. 2023 Oct 31;28(10):106004. doi: 10.1117/1.JBO.28.10.106004 (PMC10617155; doi:10.1117/1.JBO.28.10.106004)
Supplement: Supplementary file 1 [file JBO_028_106004_SD001.pdf]

Rater	Minimal detectable PpIX concentration		
	Microscope	Loupe Device - Low Background Illumination Mode	Loupe Device - High Background Illumination Mode
Rater #1	16µg/ml	8µg/ml	12µg/ml
Rater #2	17µg/ml	8µg/ml	11µg/ml
Rater #3	15µg/ml	8µg/ml	12µg/ml
Rater #4	16µg/ml	8µg/ml	12µg/ml
Rater #4	15µg/ml	7µg/ml	10µg/ml
